# Percentage of Low-Attenuation Epicardial Adipose Volume (LAAV%) as an Independent Predictor of Coronary Microvascular Dysfunction: A Retrospective Cohort Study

**DOI:** 10.31083/RCM50334

**Published:** 2026-08-05

**Authors:** Hui Ding, Linlin Sun, Shuangxiang Lin, Xincheng Li, Haipeng Liu, Zhen Wang

**Affiliations:** ^1^Department of Radiology, Zhejiang Chinese Medical University, 310053 Hangzhou, Zhejiang, China; ^2^Department of Radiology, Affiliated Zhejiang Hospital, Zhejiang University School of Medicine, 310013 Hangzhou, Zhejiang, China; ^3^Department of Radiology, The Fourth School of Clinical Medicine, Zhejiang Chinese Medical University, Hangzhou First People’s Hospital, 310006 Hangzhou, Zhejiang, China; ^4^Universidad Santa Paula, 11803 Curridabat, San José Province, Costa Rica; ^5^Cardiovascular Analytics Group, Hong Kong Special Administrative Region, China

**Keywords:** coronary microvascular dysfunction, adipose tissue, epicardial, computed tomography angiography, low-attenuation adipose tissue, perivascular inflammation

## Abstract

**Background:**

Clinically, coronary microvascular dysfunction (CMD) remains a significant diagnostic challenge because the associated symptoms frequently overlap with obstructive coronary artery disease (CAD). This condition may contribute to cardiac ischemic events regardless of the presence of obstructive epicardial lesions. While inflammatory pathways, particularly those involving epicardial adipose tissue (EAT) accumulation, play a central role in the development of CMD, the specific influence of EAT quality, as reflected in the associated density-based composition, remains poorly understood.

**Methods:**

This retrospective study (n = 155) analyzed a single primary vessel per patient to ensure independence of data. Based on invasive physiological assessment, patients were stratified into an isolated CMD group (n = 81), defined as fractional flow reserve (FFR) ≥0.75 with coronary flow reserve (CFR) <2.5 and/or index of microcirculatory resistance (IMR) ≥25 and a non-CMD control group (n = 74), which included patients with obstructive CAD (FFR <0.75, stenosis >50%) and those with normal physiology. Quantitative assessment using coronary computed tomography angiography (CCTA) applied a range of –190 to –15 Hounsfield units (HU). Perivascular inflammation was evaluated by measuring the low-attenuation adipose volume (LAAV) (<–75 HU) as a percentage of total EAT (LAAV%). Independent determinants of CMD were identified using multivariable logistic regression models.

**Results:**

Multivariable analysis identified standardized LAAV% as the primary independent predictor of CMD. Specifically, each one-standard deviation (SD) increase in LAAV% was associated with a 147.1% increase in odds of CMD (odds ratios (OR) = 2.471, 95% confidence interval (CI): 1.577–4.128, *p* < 0.001). The predictive framework demonstrated robust performance (area under the receiver operating characteristic curve (AUC) = 0.810). Additional significant risk factors included body mass index (BMI) (OR = 1.380) and smoking history (OR = 4.005, *p* = 0.023).

**Conclusions:**

Low-attenuation areas of EAT, particularly as quantified by standardized LAAV%, are strong independent predictors of CMD. The integration of LAAV% into non-invasive risk assessments may improve diagnostic accuracy and help inform targeted interventions for CMD.

## 1. Introduction

Non-obstructive coronary syndromes, including ischemia with non-obstructive coronary arteries (INOCA), myocardial infarction with non-obstructive coronary arteries (MINOCA), and angina with non-obstructive coronary arteries (ANOCA), present significant diagnostic challenges due to the absence of flow-limiting epicardial stenosis [1]. These entities require precise ‘working diagnoses’ through advanced imaging to distinguish microvascular impairment from anatomical findings [2,3]. Impacting nearly half of the individuals within the INOCA cohort, coronary microvascular dysfunction (CMD) represents a widespread clinical condition that nevertheless remains frequently under-recognized. Even without obstructive epicardial lesions, CMD patients face elevated risks for major adverse cardiac events, encompassing myocardial infarction and heart failure [4]. Indeed, the five-year risk of major adverse cardiovascular events (MACE) in this population is more than double that of patients without CMD.

Located within the anatomical space between the pericardium and myocardium, epicardial adipose tissue (EAT) represents a unique visceral adipose depot sharing an immediate paracrine link with the underlying myocardium, unhindered microcirculatory interface with the heart’s musculature [5,6]. In a healthy state, the heart benefits from the cardioprotective role of EAT, which primarily involves the release of anti-inflammatory adipokines. These mediators are essential for maintaining local metabolic equilibrium and facilitating vascular expansion. However, in the presence of obesity or metabolic syndrome, EAT undergoes adverse remodeling and phenotypic shifts, transitioning into a dysfunctional, pro-inflammatory state. This dysfunction exacerbates local inflammation, which negatively affects both the myocardium and the coronary microvasculature [7,8].

Although research has shown a strong link between abnormal EAT accumulation and CMD [9], the specific connection between low-attenuation EAT and CMD remains unclear [5,10]. To exclude flow-limiting epicardial narrowing in symptomatic individuals, coronary computed tomography angiography (CCTA) is conventionally utilized as the primary non-invasive diagnostic instrument within standard clinical workflows. However, standard CCTA has only limited ability to definitively diagnose CMD, often requiring subsequent invasive physiological assessments such as coronary flow reserve (CFR) and index of microcirculatory resistance (IMR) to confirm the disease. While traditional invasive coronary angiography (ICA) is limited to the assessment of epicardial vessel anatomy, research has shown that advanced cardiac computed tomography (CT) offers a broader evaluation by combining anatomical imaging with myocardial tissue characterization. Techniques such as late iodine enhancement (LIE) and adipose tissue phenotyping uniquely enable the detection of local fibrosis and inflammation by CT. These pathophysiological drivers remain invisible to ICA [11]. Consequently, the superior ability of CT to perform detailed tissue characterization offers significant benefits for all-encompassing non-invasive assessments, even as invasive physiological testing continues to be regarded as the definitive criterion for CMD verification [12].

Previous research has shown that reduced total attenuation values for EAT are associated with microvascular dysfunction. However, whether low-attenuation adipose volume (LAAV) is independently associated with CMD remains to be determined [9,10,13]. This study leverages CCTA to quantify the LAAV% as a novel marker for CMD, consistent with emerging perspectives that position adipose imaging as a critical biomarker in non-obstructive cohorts [14]. Ultimately, this work seeks to delineate a precise diagnostic pathway for identifying individuals at high risk for CMD, potentially enabling personalized prevention strategies that target epicardial adiposity in this vulnerable population.

## 2. Methods

### 2.1 Study Population

Study enrollment spanned January 2020 to January 2025. A retrospective review was conducted on 178 consecutive patients presenting with stable chest pain and suspected microvascular dysfunction at Hangzhou First People’s Hospital (The Fourth School of Clinical Medicine, Zhejiang Chinese Medical University). Study inclusion required symptomatic patients to exhibit suspected non-obstructive coronary arteries (INOCA), characterized by objective ischemic evidence and non-obstructive epicardial arteries.

### 2.2 Patient Selection and Ethical Considerations

**Fig. 1.**
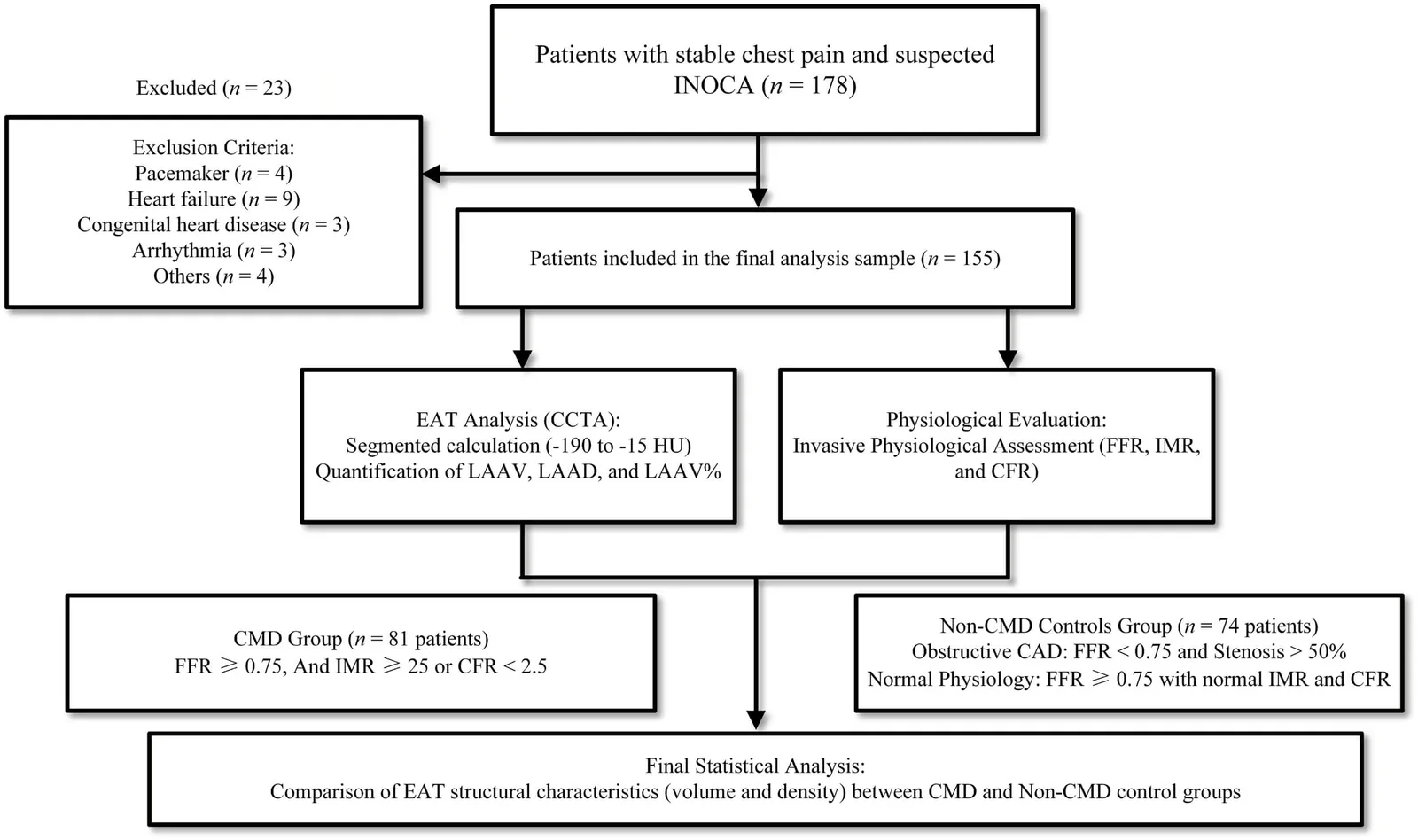
**Flowchart of patient enrollment, exclusion, and study design**. After applying the predefined exclusion criteria to the initial 178 patients with suspected INOCA, a final cohort of 155 patients (with one target vessel analyzed per patient) was included for comprehensive CCTA and invasive physiological assessment. Abbreviations: INOCA, ischemia and non-obstructive coronary arteries; CCTA, coronary computed tomography angiography; EAT, epicardial adipose tissue; LAAV, low-attenuation adipose volume; FFR, fractional flow reserve; IMR, index of microcirculatory resistance; CFR, coronary flow reserve; CMD, coronary microvascular dysfunction; HU, Hounsfield units; LAAD, low-attenuation adipose density; CAD, coronary artery disease.

Following the initial screening, 23 individuals were excluded from the study population based on predefined criteria illustrated in our flowchart (Fig. 1). The specific reasons for exclusion included a history of heart failure (*n* = 9), permanent pacemaker implantation (*n* = 4), congenital heart disease (*n* = 3), and significant cardiac arrhythmias (*n* = 3). Other clinical contraindications necessitated the exclusion of four additional patients, such as a history of cardiovascular surgery, known iodine intolerance, or severe renal impairment [15].

The final analytical cohort consisted of 155 patients. To ensure data independence, only one primary target vessel per patient was selected for comprehensive physiological and imaging assessment. The study protocol was approved by the Ethics Committee of Hangzhou First People’s Hospital (Approval No. 2025ZN097-1) on April 8, 2025. While the primary project originally investigated aortic stiffness in patients with ANOCA, the present study represents a pre-specified secondary analysis of the same CCTA datasets, specifically evaluating EAT and microvascular performance within the INOCA subgroup. Research protocols followed the Declaration of Helsinki. While participants provided general written informed consent for the potential research use of their de-identified data at the time of initial clinical evaluation, a specific waiver of individual informed consent was granted by the IRB for this retrospective analysis of the existing datasets.

### 2.3 Invasive Physiological Assessment and Grouping

Invasive coronary physiological assessments were performed on all patients using a sensor-tipped PressureWire X (Abbott Vascular, Santa Clara, CA, USA). Intravenous adenosine (140 μg/kg/min) was administered to induce sustained maximal hyperemia. CFR was measured to evaluate the hyperemic blood flow augmentation, while the IMR was calculated to quantitatively assess resistance of the coronary microvascular compartment [1,16]. Based on these precise invasive results, the 155 patients were stratified into two groups:

**CMD Group (*****n***** = 81)**: This group included patients with hemodynamically non-significant epicardial stenosis fractional flow reserve (FFR) ≥0.75 and objective microvascular impairment (IMR ≥25, CFR <2.5 or both) [5,17].

**Control Group (*****n *****= 74)**: This group served as a non-isolated CMD reference and was comprised of individuals who failed to meet the stringent diagnostic criteria for primary CMD. It included patients with either obstructive coronary artery disease (CAD) (FFR <0.75 and angiographic stenosis >50%)—where microvascular indices are potentially confounded by epicardial flow restriction—or those with entirely normal coronary physiology (normal FFR, IMR, and CFR values).

### 2.4 CCTA Acquisition and Reconstruction

All CCTA datasets were obtained via a dual-source CT platform (Somatom Definition Flash; Siemens Healthcare, Forchheim, Germany). A standard non-ionic iodinated contrast medium with an iodine concentration of 350–370 mg I/mL was administered intravenously for CCTA acquisition. To ensure optimal image quality across varying heart rates, scanning modes were selected according to heart rate: prospective electrocardiogram (ECG)-triggering (stable/low rates) or retrospective ECG-gating (high/irregular rhythms). Superior temporal resolution (dual-source system) bypassed pre-scan beta-blocker requirements. Radiation protocols (as low as reasonably achievable (ALARA) principle) integrated automated current modulation and body mass index (BMI)-adjusted tube voltage (100–120 kVp). The mean effective radiation dose was 3.2 ± 1.1 mSv for CCTA and 6.5 ± 2.4 mSv for ICA. This dual-modality protocol was clinically justified as ICA provided the diagnostic gold standard for invasive physiological assessment (IMR and CFR), whereas CCTA enabled the non-invasive, high-resolution phenotyping of EAT characteristics, which is not feasible through invasive angiography alone. Image datasets were reconstructed at 0.75 mm thickness; 0.5 mm increment, ensuring high spatial resolution for detailed anatomical characterization. The reconstructed datasets were subsequently transferred to the Syngo.VIA workstation (version VB20; Siemens Healthineers, Erlangen, Germany) for post-processing [18,19].

### 2.5 EAT Quantification and ROI Definition

Two blinded radiologists (independent; unaware of clinical or invasive findings) quantified EAT comprises the adipose deposit intervening between the myocardium and visceral pericardium. A semi-automatic threshold segmentation technique (Syngo.VIA) was utilized to calculate volumes based on objective Hounsfield units (HU) ranges. Within this standardized workflow, the primary role of the radiologists was to perform anatomical boundary verification and systematic auditing of the regions of interest (ROI) to ensure the accuracy and integrity of the automated process.

Utilizing HU values, we stratified EAT according to established radiological literature. The chosen window (–190 to –15 HU) parallels validated contrast-enhanced cardiac imaging criteria, maximizing sensitivity for perivascular adipose tissue while mitigating potential attenuation shifts. Total EAT volume (EFV) was defined as –190 to –15 HU; LAAV was defined as –190 to –75 HU, a range conventionally characterized in the literature as a metabolically active and “pro-inflammatory” phenotype; Medium-attenuation adipose volume (MAAV) as –75 to –45 HU; and High-attenuation Adipose volume (HAAV) as –45 to –15 HU [19,20]. To account for variations in body size, the following indices were calculated: EFV adjusted by body surface area (BSA) (EFVi = EFV/BSA), LAAV% (LAAV/EFV × 100%), and indexed low-attenuation adipose volume (LAAVi) (LAAV/BSA) [10].

### 2.6 Clinical Data Collection

Baseline clinical and demographic data were systematically collected for all participants, allowing thorough characterization of the study population. Initial clinical evaluation focused on baseline characteristics, encompassing age, sex, and BMI for anthropometric profiling. Clinical assessment encompassed baseline comorbidities and cardiovascular risk factors (hypertension; diabetes mellitus; smoking history; serum lipid profiles) [21].

### 2.7 Statistical Analysis

Normality assessment utilized the Shapiro-Wilk test. Variables are reported by distribution: mean ± standard deviation (SD), and non-normally distributed continuous variables as the median with interquartile range (IQR). Categorical variables appear as absolute frequencies (percentages). Univariable group comparisons (normally distributed data) utilized the independent Student’s *t*-test, non-normally distributed variables using the Mann-Whitney U test, and categorical variables using the Pearson *χ*^2^ test or Fisher’s exact test, as appropriate. Effect sizes for continuous variables were evaluated using Cohen’s *d*, with values of 0.20, 0.50, and 0.80 indicating small, moderate, and large effects, respectively. Two-tailed testing was used for all comparisons, with the significance threshold set at *p *< 0.05.

For preliminary screening, the discriminative power of continuous EAT-related indices across groups (Table 3) was evaluated by Analysis of Variance (ANOVA). The F-statistic and partial eta-squared (*η_p_*^2^) were calculated to determine their respective effect sizes. Univariable logistic regression estimated unadjusted odds ratios (OR) to screen for potential CMD predictors. Multivariable logistic regression incorporated variables demonstrating univariable significance (*p* < 0.10). To strictly adhere to the events-per-variable (EPV) principle and avoid model overfitting given the sample size, a parsimonious multivariable model was constructed. Using a forced entry method, this model adjusted for age, sex, BMI, smoking history, glycated hemoglobin (HbA1c), and triglycerides (TGs). Highly collinear variables such as LAAVi and total EFV were excluded prior to model fitting to prevent multicollinearity. This was further confirmed by variance inflation factor (VIF) values <5 [9,10,22].

Adjusted OR with 95% confidence intervals (CIs) quantified associations. The Hosmer-Lemeshow test verified model calibration. A Spearman correlation matrix, organized by clinical categories (e.g., epicardial fat, demographics, and metabolism), was constructed to visualize variable interrelationships. To identify distinct clinical phenotypes, unsupervised K-means clustering was performed based on EAT indices and metabolic parameters, with stability validated by the Adjusted Rand Index (ARI). Diagnostic performance for identifying high-risk phenotypes (Cluster 2) was evaluated via receiver operating characteristic (ROC) analysis. Area under the curve (AUC) values were compared using the DeLong test in the ‘pROC’ package. The intraclass correlation coefficient (ICC) determined both intra- and inter-observer reliability. All analyses were conducted using R (version 4.5.1; R Foundation for Statistical Computing, Vienna, Austria, including the 'rms' package for 4-knot restricted cubic spline analysis) and SPSS (version 29.0; IBM Corp., Armonk, NY, USA) [23,24].

## 3. Results

### 3.1 Study Population and Baseline Characteristics.

The detailed participant enrollment and exclusion process are illustrated in the study flowchart (Fig. 1). The final analytical cohort comprised 155 patients, categorized into the CMD group (*n* = 81) and the control group (*n* = 74). Baseline demographic characteristics, including age and sex distribution, were comparable between the two groups (*p* > 0.05). However, patients in the CMD group exhibited significantly higher BMI (25.1 ± 2.6 vs. 23.2 ± 2.2 kg/m^2^) and TG levels [1.76 (1.53–2.08) vs. 1.31 (1.17–1.58) mmol/L] compared to the control group (*p* = 0.001). Baseline participant characteristics—clinical and demographic are outlined in Table 1.

**Table 1.** **Baseline characteristics and univariate analysis of risk factors for CMD**.

Parameters		Whole cohort (*n* = 155)	CMD group (*n* = 81)	Non-CMD (*n* = 74)	Difference/ OR (95% CI)	*p*-value
Demographics						
	Age, (years)	65.02 ± 9.96	64.81 ± 9.41	65.24 ± 10.59	–0.43 (–3.23, 2.41)	0.791
	Sex, male, n (%)	88 (56.8)	46 (56.8)	42 (56.8)	1.00 (0.53–1.89)	0.997
	Height, (m)	1.63 ± 0.08	1.63 ± 0.08	1.65 ± 0.07	–0.02 (–0.04, 0.00)	0.073
	Weight, (kg)	65.01 ± 9.51	66.64 ± 9.22	63.23 ± 9.56	3.41 (1.58, 5.24)	**0.019**
	BMI, (kg/m^2^)	24.22 ± 2.56	25.13 ± 2.58	23.23 ± 2.15	1.90 (1.38, 2.52)	**<0.001**
	BSA, (m^2^)	1.72 ± 0.16	1.73 ± 0.15	1.70 ± 0.16	0.03 (–0.01, 0.07)	0.180
Comorbidities, n (%)						
	Hypertension	95 (61.3)	50 (61.7)	45 (60.8)	1.04 (0.54–1.99)	0.907
	Diabetes mellitus	25 (16.1)	13 (16.0)	12 (16.2)	0.99 (0.42–2.33)	0.977
	Smoking history	30 (19.4)	20 (24.7)	10 (13.5)	2.09 (0.91–4.84)	0.103
	Myocardial bridge	49 (31.6)	31 (38.3)	18 (24.3)	1.96 (0.96–4.00)	0.061
Laboratory findings						
	FBG, (mmol/L)	5.05 (4.66–5.62)	5.04 (4.61–5.62)	5.05 (4.74–5.55)	0.01 (–0.12, 0.10)	0.773
	HbA1c, (%)	5.55 (5.40–5.78)	5.60 (5.50–5.87)	5.50 (5.40–5.70)	0.10 (–0.20, 0.40)	0.411
	TC, (mmol/L)	4.26 ± 1.02	4.32 ± 1.05	4.19 ± 0.98	0.13 (–0.14, 0.40)	0.395
	TG, (mmol/L)	1.54 (1.34–1.83)	1.76 (1.53–2.08)	1.31 (1.17–1.58)	0.45 (0.18, 0.72)	**0.001**
	HDL, (mmol/L)	1.13 ± 0.29	1.11 ± 0.28	1.15 ± 0.29	–0.04 (–0.13, 0.05)	0.380
	LDL, (mmol/L)	2.38 ± 0.79	2.46 ± 0.81	2.29 ± 0.75	0.17 (–0.06, 0.40)	0.155
	CRP, (mg/L)	2.93 (2.48–3.33)	3.30 (2.75–3.60)	2.55 (2.20–3.05)	0.75 (–0.02, 1.52)	0.067
	FFA, (μmol/L)	404.71 ± 217.68	389.39 ± 216.72	421.47 ± 219.55	–32.08 (–99.73, 35.57)	0.359
	FIB, (g/L)	2.89 (2.70–2.98)	2.94 (2.70–3.09)	2.85 (2.70–2.93)	0.09 (–0.19, 0.37)	0.903
	EF (M/2D) (%)	65.33 ± 7.01	65.55 ± 6.55	65.08 ± 7.53	0.47 (–1.54, 2.48)	0.556

Note: Values are expressed as the mean ± SD, median (interquartile range), or *n* (%). Normality of data distribution was evaluated using the Shapiro-Wilk test. Continuous variables were compared using Student’s *t*-test or the Mann-Whitney U test, as appropriate; categorical variables were compared using the *χ*^2^ test or Fisher’s exact test. The penultimate column reports mean differences for normally distributed continuous variables, location differences for non-normally distributed continuous variables, and unadjusted odds ratios (ORs) with 95% confidence intervals (CIs) for binary categorical variables. Bold text indicates statistical significance at *p* < 0.05. Abbreviations: BSA, body surface area; BMI, body mass index; CRP, C-reactive protein; CI, confidence interval; EF, ejection fraction; FFA, free fatty acids; FBG, fasting blood glucose; FIB, fibrinogen; HbA1c, glycated hemoglobin; HDL, high-density lipoprotein; LDL, low-density lipoprotein; TC, total cholesterol; TG, triglycerides; OR, odds ratio; SD, standard deviation.

### 3.2 CCTA-Derived EAT Characteristics

**Fig. 2.**
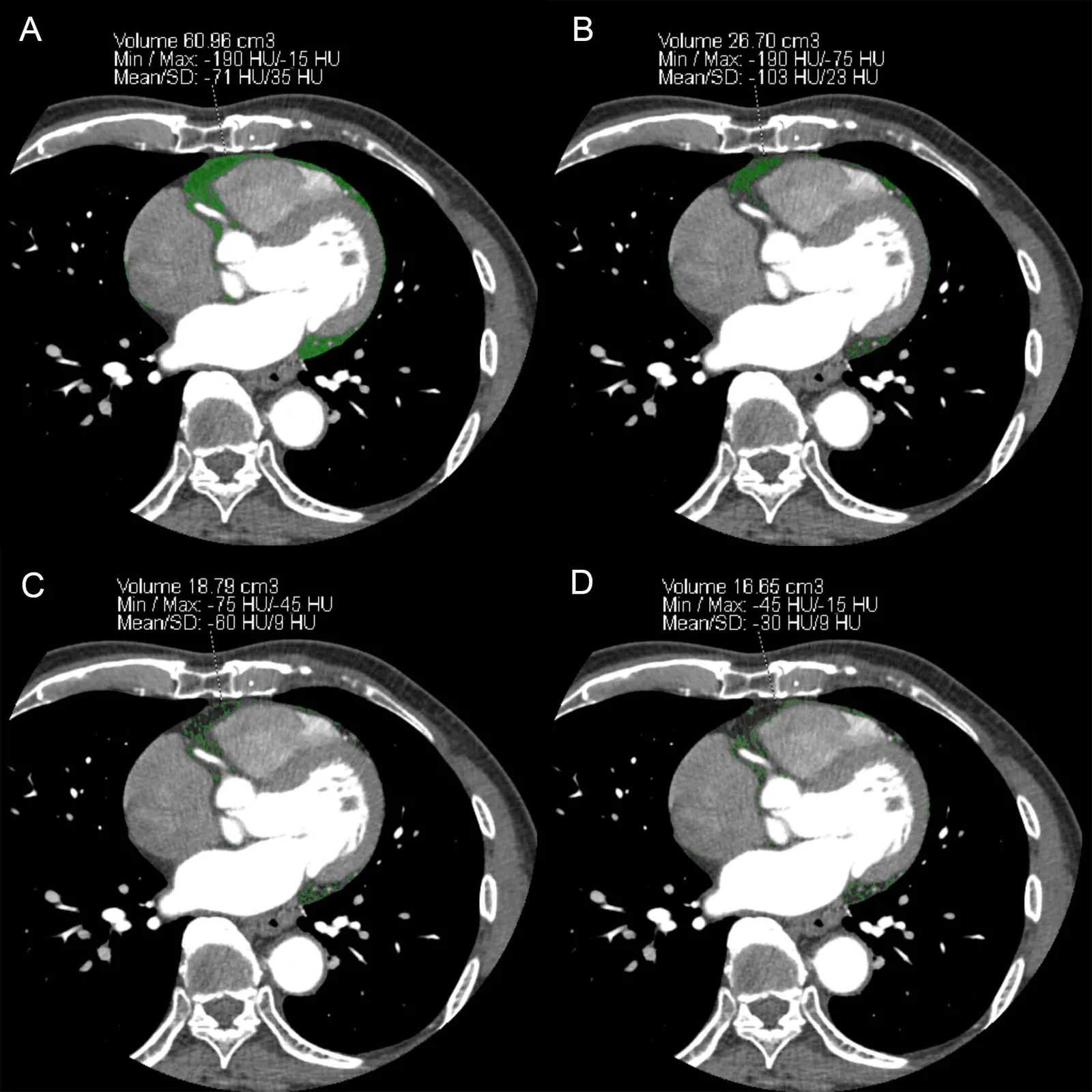
**Illustrative CCTA slices for quantifying EAT volumes and densities**. (A) EFV segmented from –190 to –15 HU. (B) LAAV from –190 to –75 HU. (C) MAAV from –75 to –45 HU. (D) HAAV from –45 to –15 HU. Abbreviations: CCTA, coronary computed tomography angiography; EAT, epicardial adipose tissue; HU, Hounsfield units; EFV, epicardial adipose volume; LAAV, low-attenuation adipose volume; MAAV, medium-attenuation adipose volume; HAAV, high-attenuation adipose volume.

**Fig. 3.**
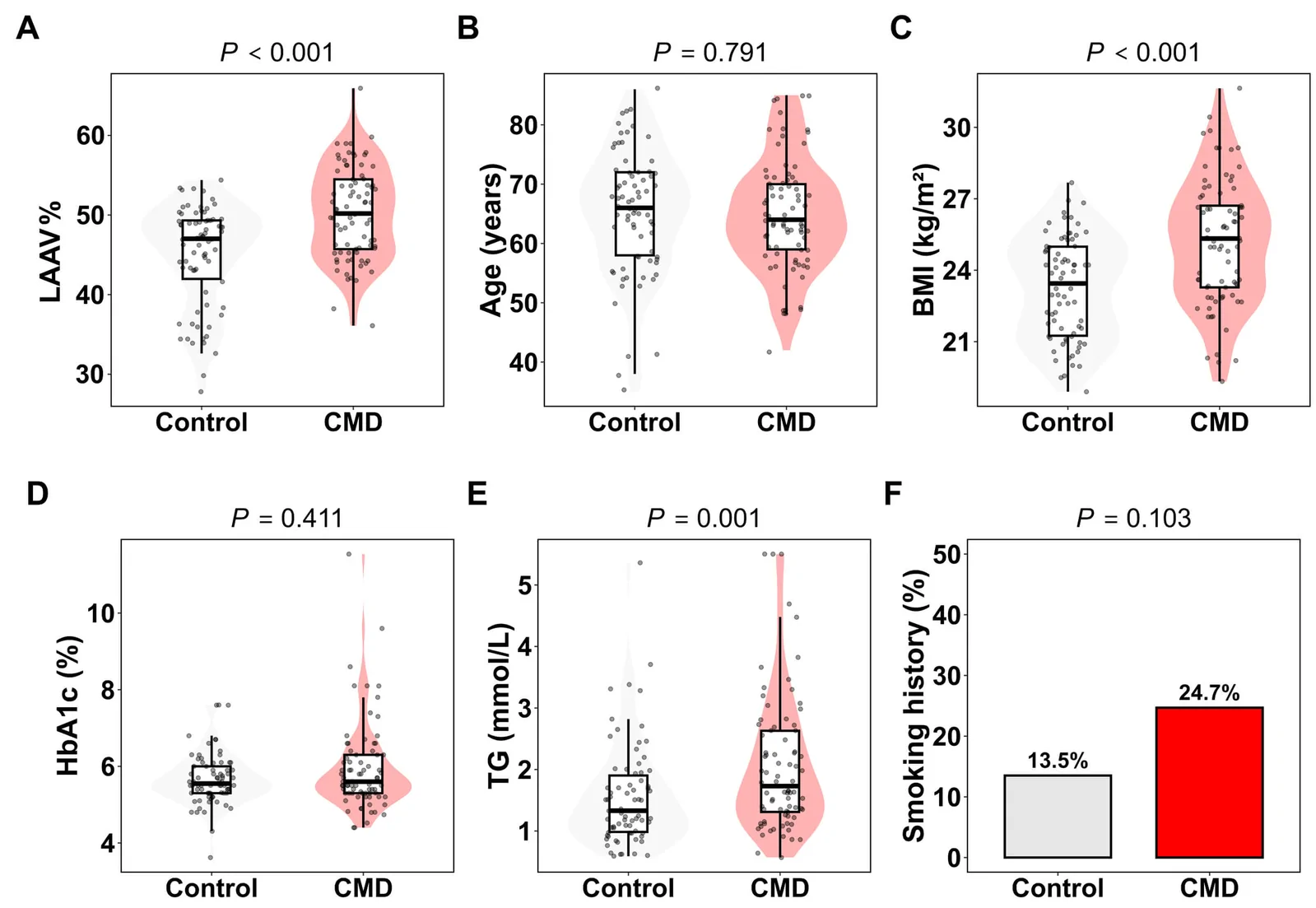
**Distribution of EAT characteristics and clinical parameters between groups. Comparison of (A) LAAV%, (B) **a**ge, (C) BMI, (D) HbA1c, (E) triglycerides, and (F) smoking history rate between the CMD and non-CMD groups. **Box and violin plots show median, interquartile range, and distribution density; bar chart shows proportion. Abbreviations: BMI, body mass index; CMD, coronary microvascular dysfunction; EAT, epicardial adipose tissue; HbA1c, glycated hemoglobin; LAAV%, percentage of low-attenuation adipose volume; TG, triglycerides.

CCTA was used to quantify EAT subgroups, with representative examples of the semi-automatic segmentation shown in Fig. 2. EFV, epicardial adipose volume index (EFVi), and LAAV were significantly elevated in the CMD cohort relative to non-CMD controls (*p* = 0.029, *p* = 0.038, and *p* < 0.001, respectively; Table 2). Notably, the CMD group also demonstrated a markedly higher LAAV% (*p* < 0.001), whereas HAAV% was lower (*p* = 0.002). These distribution patterns and the significant differences in LAAV% are visualized in Fig. 3. Given its stronger statistical association with CMD in univariate analysis, LAAV% was selected as the primary EAT component for subsequent multivariate modeling.

**Table 2.** **CCTA-derived epicardial adipose tissue characteristics**.

Parameters		CMD group (*n *= 81)	Non-CMD (*n* = 74)	Mean diff (95% CI)	*p*-value	Cohen’s d (95% CI)
EAT volume						
	LAAV, (cm^3^)	39.43 ± 10.49	32.59 ± 11.93	6.84 (4.12, 9.56)	**<0.001**	0.60 (0.29, 0.91)
	MAAV, (cm^3^)	20.99 ± 4.09	20.13 ± 5.36	0.86 (–0.66, 2.38)	0.291	0.18 (–0.14, 0.50)
	HAAV, (cm^3^)	18.55 ± 3.21	18.10 ± 4.44	0.45 (–0.27, 1.17)	0.221	0.12 (–0.20, 0.44)
	EFV, (cm^3^)	77.76 ± 15.85	71.16 ± 21.29	6.60 (2.08, 11.12)	0.029	0.35 (0.03, 0.67)
	EFVi, (cm^3^/m^2^)	44.92 ± 8.42	41.73 ± 7.22	3.19 (1.25, 5.13)	0.038	0.41 (0.09, 0.73)
	LAAVi, (cm^3^/m^2^)	22.76 ± 5.62	19.11 ± 6.29	3.65 (1.75, 5.56)	**<0.001**	0.61 (0.28, 0.93)
	HAAVi, (cm^3^/m^2^)	10.73 ± 1.72	10.65 ± 2.22	0.08 (–0.38, 0.54)	0.736	0.04 (–0.27, 0.36)
EAT density						
	LAAD, (HU)	–107.28 ± 2.21	–106.22 ± 2.28	–1.06 (–1.78, –0.34)	**0.004**	–0.47 (–0.78, –0.16)
	MAAD, (HU)	–60.35 ± 0.53	–60.12 ± 0.60	–0.23 (–0.39, –0.07)	**0.006**	–0.41 (–0.73, –0.09)
	HAAD, (HU)	–30.09 ± 0.60	–30.08 ± 0.61	–0.01 (–0.19, 0.17)	0.918	–0.02 (–0.33, 0.30)
	EFD, (HU)	–76.90 ± 4.45	–73.65 ± 5.76	–3.25 (–4.72, –1.78)	**<0.001**	–0.64 (–0.96, –0.31)
EAT distribution						
	LAAV%	50.27 ± 5.73	44.96 ± 6.35	5.31 (3.78, 6.84)	**<0.001**	0.88 (0.55, 1.21)
	HAAV%	24.24 ± 3.48	26.26 ± 4.90	–2.02 (–3.24, –0.80)	**0.002**	–0.47 (–0.79, –0.16)

Values are expressed as the mean ± SD. Abbreviations: CI, confidence interval; EAT, epicardial adipose tissue; EFD, epicardial adipose density; EFV, epicardial adipose volume; EFVi, epicardial adipose volume index; LAAD, low-attenuation adipose density; LAAV, low-attenuation adipose volume; LAAVi, indexed low-attenuation adipose volume; LAAV%, percentage of low-attenuation adipose volume; MAAV, medium-attenuation adipose volume; MAAD, medium-attenuation adipose density; HAAV, high-attenuation adipose volume; HAAD, high-attenuation adipose density; HAAVi, indexed high-attenuation adipose volume; CMD, coronary microvascular dysfunction. EAT Definition: Attenuation ranges: Low-density (–190 to –75 HU); Middle-density (–75 to –45 HU); High-density (–45 to –15 HU). The impact of the observed differences was categorized via Cohen’s *d* values, following established criteria for minor (0.20), moderate (0.50), and substantial (0.80) effects. Bold entries signify *p* < 0.05.

### 3.3 Screening of EAT-Related Predictors

**Fig. 4.**
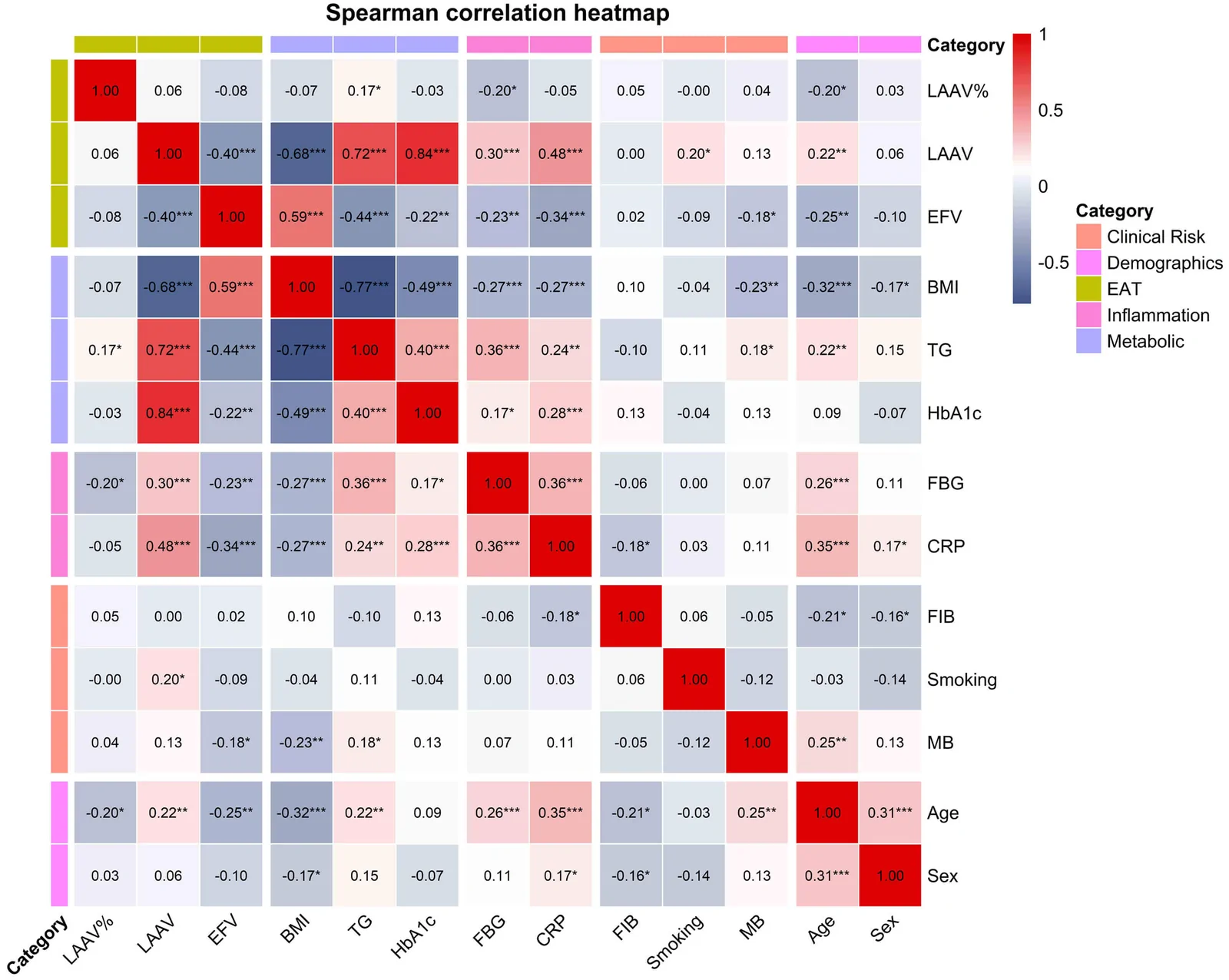
**Correlation matrix of EAT metrics and clinical parameters**. Spearman’s rank correlation (*ρ*) showing associations between CCTA-derived EAT phenotypes and clinical/laboratory parameters. Red and blue indicate positive and negative correlations, respectively. *p < 0.05, **p < 0.01, ***p < 0.001. Abbreviations: BMI, body mass index; CRP, C-reactive protein; EAT, epicardial adipose tissue; EFV, epicardial adipose volume; FBG, fasting blood glucose; FIB, fibrinogen; HbA1c, glycated hemoglobin; LAAV, low-attenuation adipose volume; LAAV%, percentage of LAAV; MB, myocardial bridging; TG, triglycerides.

Associations between EAT subcomponents and common clinical risk markers are presented in the heatmap (Fig. 4). To identify the most robust imaging markers for CMD, we performed univariate screening to evaluate the discriminative power of each EAT parameter (Table 3). Among all metrics, LAAV% exhibited the highest F-statistic (29.90) and the largest partial *η*^2^ (0.163), identifying it as the strongest imaging predictor for the presence of CMD. Consequently, LAAV% was prioritized for inclusion in the subsequent multivariate logistic regression models.

**Table 3.** **Univariate analysis of EAT-related indices for CMD screening**.

Variables	*F*-value	*p*-value	Partial* η*^2^	Interpretation
LAAV	14.09	**<0.001**	0.084	High significance
LAAD	6.16	**0.014**	0.039	Moderate significance
EFD	15.60	**<0.001**	0.093	High significance
EFV	4.845	**0.029**	0.031	Moderate significance
LAAV%	29.90	**<0.001**	0.163	Strongest predictor
LAAVi	14.28	**<0.001**	0.085	High significance

Note: All variables were compared using one-way analysis of variance (ANOVA). Effect size was evaluated using partial eta-squared (partial *η*^2^), which measures the proportion of variance in CMD risk attributable to each variable (effect size threshold: ≥0.14 indicates a strong effect). LAAV% exhibited the highest F-statistic and the largest effect size (partial *η*^2^ = 0.163), identifying it as the strongest predictor among the EAT-related indices. Bold entries signify *p* < 0.05. EFD, epicardial adipose density; EFV, epicardial adipose volume; LAAD, low-attenuation adipose density; LAAV, low-attenuation adipose volume; LAAVi, indexed low-attenuation adipose volume.

### 3.4 Multivariable Predictors of CMD

**Fig. 5.**
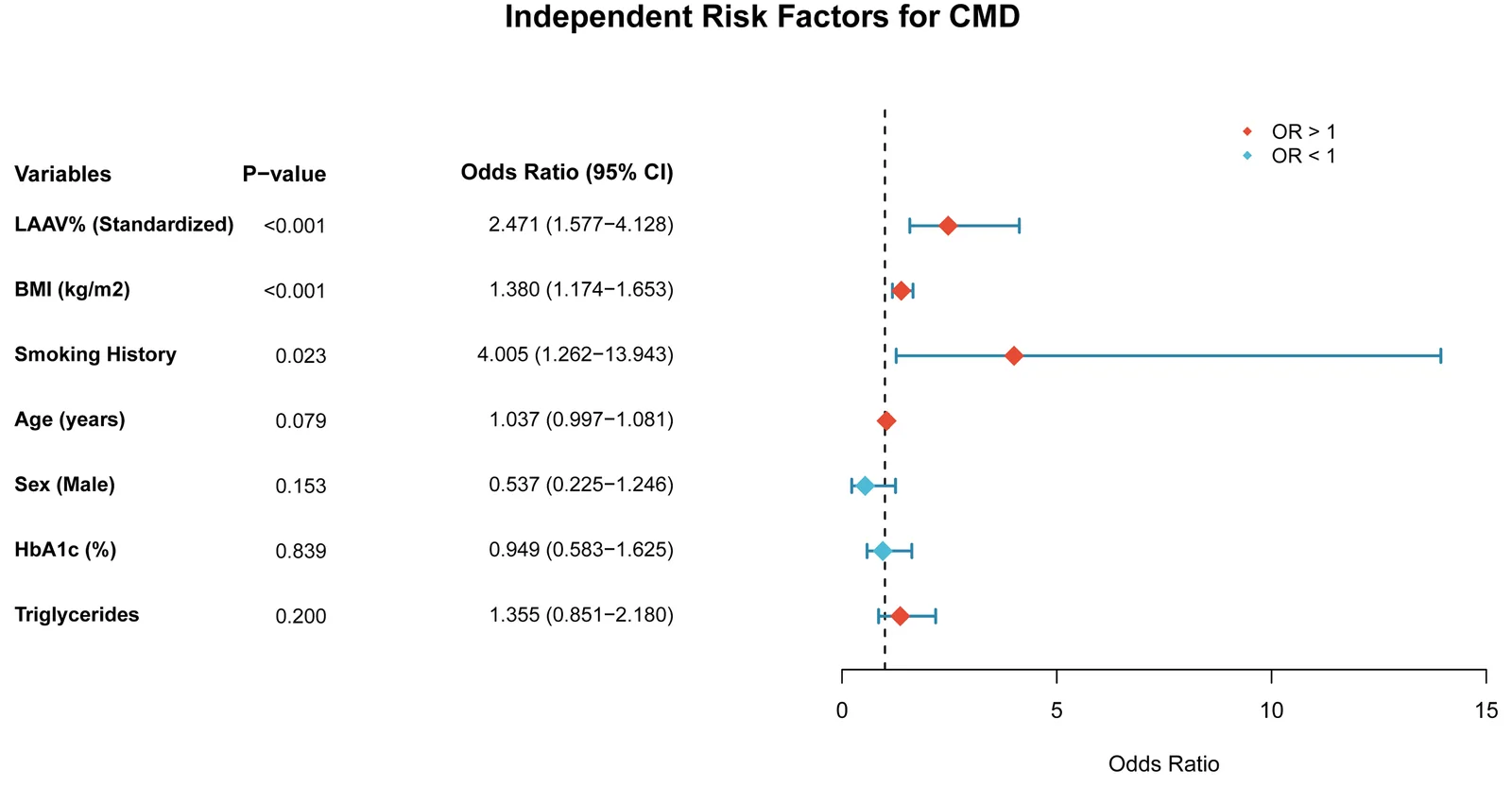
**Forest plot of independent risk factors for CMD**. The plot presents the adjusted odds ratios (ORs) and corresponding 95% confidence intervals (CIs) for predictors identified in the parsimonious multivariable logistic regression model. Notably, the OR for LAAV% is standardized per standard deviation (SD) increase to allow for direct comparison of effect sizes among predictors, while clinical factors (e.g., BMI and smoking history) are reported per unit increase. The vertical dashed line at OR = 1 indicates the null reference value. Red diamonds represent point estimates with OR >1, while blue diamonds represent point estimates with OR <1. LAAV, low-attenuation adipose volume; BMI, body mass index; CMD, coronary microvascular dysfunction; HbA1c, glycated hemoglobin.

We next performed multivariable logistic regression to adjust for potential confounding variables and identify independent predictors of CMD (Table 4). Our analysis identified standardized LAAV% as the strongest independent predictor of CMD: for every one-SD increase in LAAV%, the risk of CMD increased by approximately 147.1% (OR: 2.471; 95% CI: 1.577–4.128; *p* < 0.001). BMI was also identified as a major independent risk factor (OR: 1.380; 95% CI: 1.174–1.653; *p* < 0.001), with the probability of CMD exceeding 50% once a patient’s BMI surpassed 28 kg/m^2^. Furthermore, Smoking history was another significant independent risk factor for CMD (OR: 4.005; 95% CI: 1.262–13.943; *p* = 0.023). These predictors, which strictly adhere to the events-per-variable principle, are summarized in the forest plot (Fig. 5).

**Table 4.** **Independent determinants of CMD identified by multivariable logistic modeling**.

Variables	*OR* (95% CI)	*p*-value
LAAV% (standardized)	2.471 (1.577–4.128)	**<0.001**
BMI (kg/m^2^)	1.380 (1.174–1.653)	**<0.001**
Smoking history	4.005 (1.262–13.943)	**0.023**
Age, (years)	1.037 (0.997–1.081)	0.079
Sex, (male)	0.537 (0.225–1.246)	0.153
HbA1c (%)	0.949 (0.583–1.625)	0.839
Triglycerides (mmol/L)	1.355 (0.851–2.180)	0.200

Note: Independent CMD predictors were identified via a parsimonious multivariable logistic regression framework employing the enter technique. The model was refined to include seven core variables to ensure adherence to the events-per-variable (EPV) principle and to minimize the risk of overfitting. Abbreviations: CI, confidence interval; OR, odds ratio; LAAV%, percentage of low-attenuation adipose volume; BMI, body mass index; HbA1c, glycated hemoglobin; CMD, coronary microvascular dysfunction. Statistical Notes: The model includes age, sex, BMI, smoking history, HbA1c, and triglycerides. Notably, the OR for LAAV% was standardized per standard deviation (SD) increase to allow for direct comparison of effect sizes among predictors, while other clinical variables are reported per unit increase. Boldface type denotes values that achieved statistical significance at *p* < 0.05.

### 3.5 Model Performance and Nonlinear Risk Trends

**Fig. 6.**
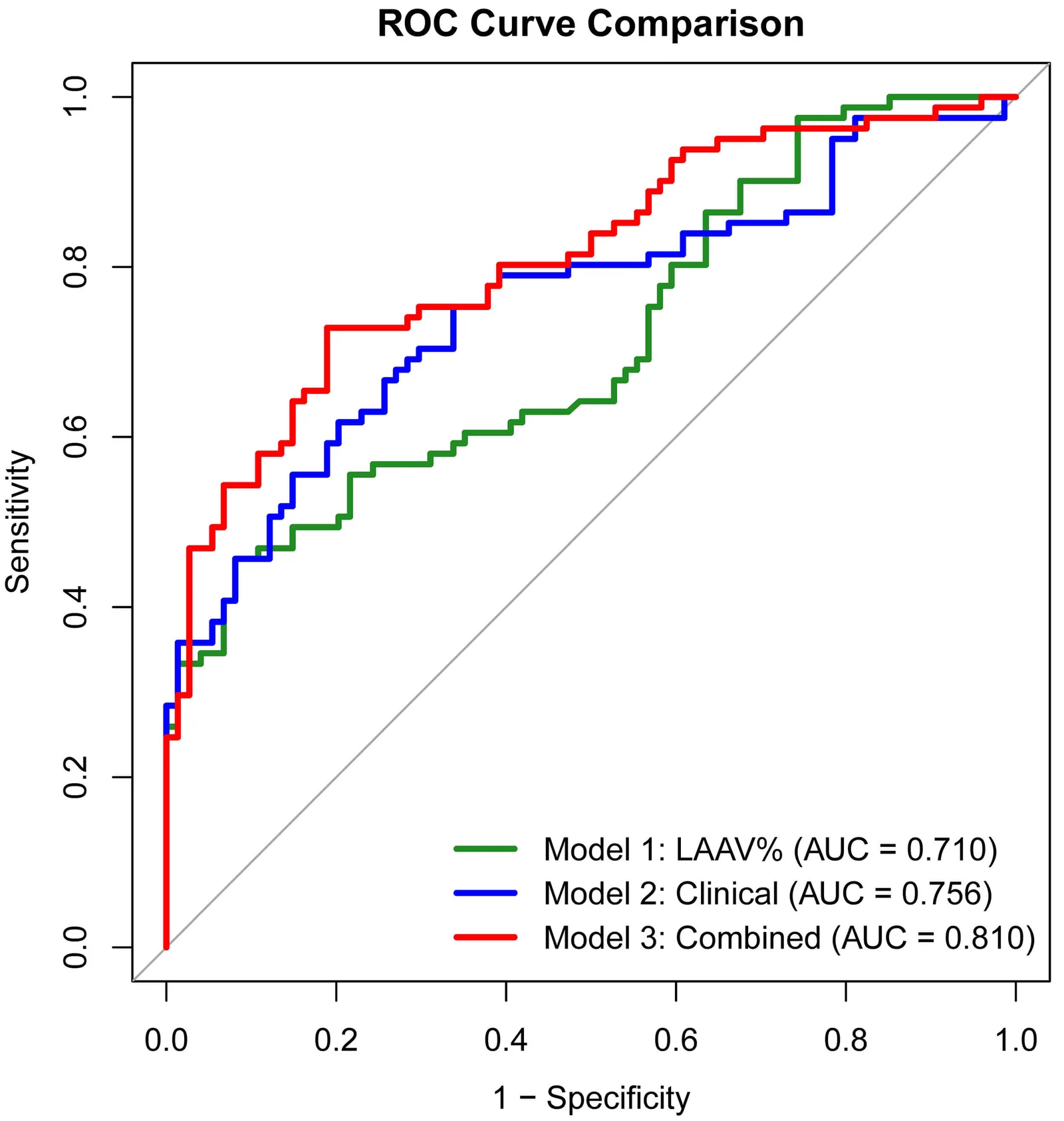
**ROC analysis for predicting CMD: comparative diagnostic performance of incremental predictive models**. The ROC curves illustrate the accuracy of three models in identifying coronary microvascular dysfunction (CMD). Model 1 includes LAAV% (AUC = 0.710). Model 2 includes clinical risk factors (AUC = 0.756). Model 3 incorporates both (AUC = 0.810, *p* < 0.05 vs. Model 1 and Model 2). These results identify LAAV% as a superior, independent imaging biomarker for CMD. Abbreviations: AUC, area under the curve; CMD, coronary microvascular dysfunction; EAT, epicardial adipose tissue; LAAV%, percentage of low-attenuation adipose volume.

**Fig. 7.**
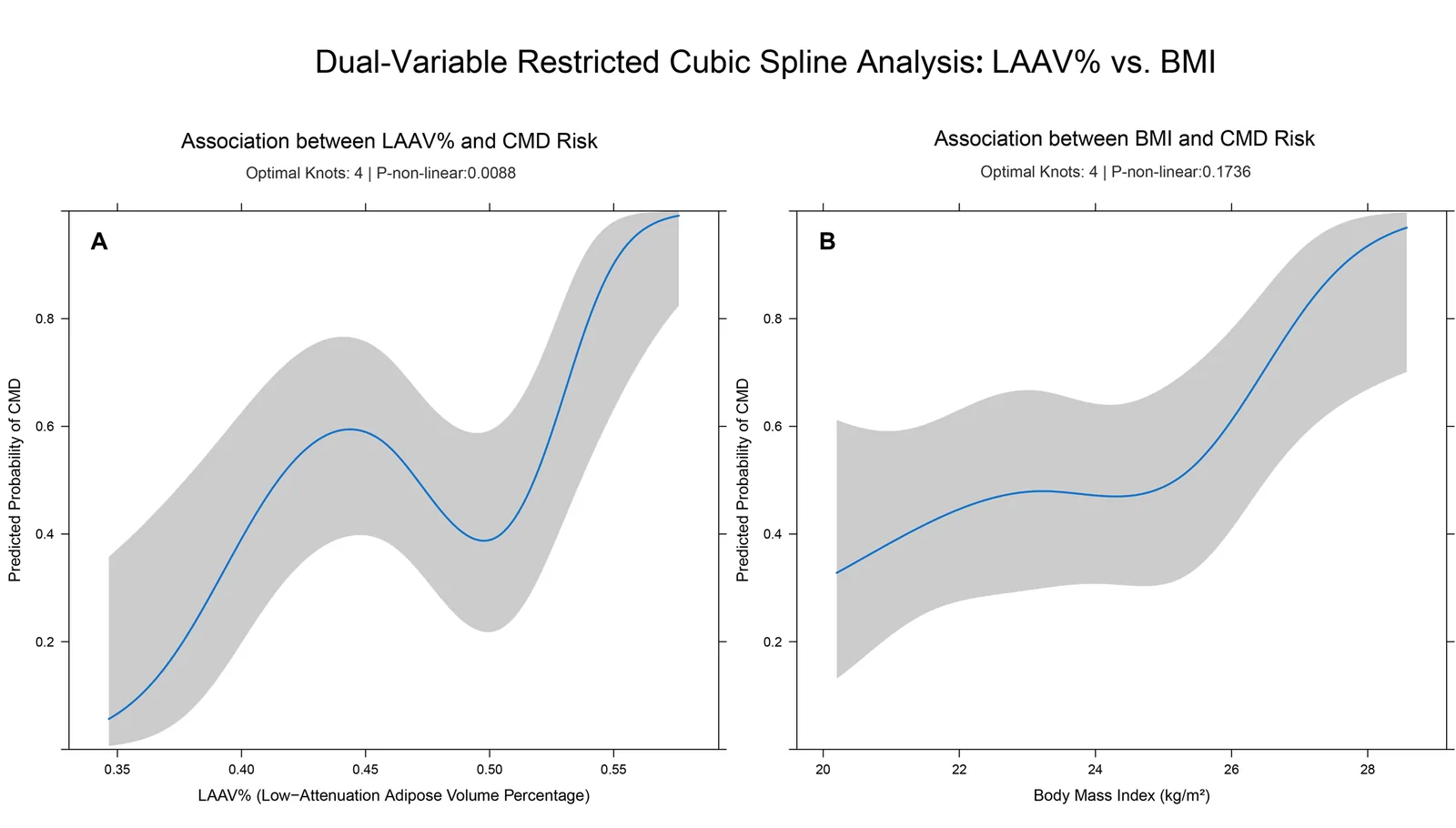
**RCS analysis reveals a non-linear relationship between LAAV% and BMI with the risk of CMD**. Restricted cubic spline (RCS) curves illustrate the predicted probability of CMD according to (A) LAAV% and (B) BMI. For LAAV%, a distinct J-shaped association is observed, with a biological ‘tipping point’ is identified at 41.7% (calculated via RCS model), beyond which the risk of CMD increases exponentially. For BMI, the RCS curve shows the dose-response relationship with CMD risk. (See Fig. 6 for corresponding diagnostic performance). Abbreviations: CMD, coronary microvascular dysfunction; LAAV, low-attenuation adipose volume; BMI, body mass index.

The diagnostic performance of the hybrid model (Model 3: Clinical + LAAV%) was superior to that of LAAV% alone (Model 1) or clinical factors alone (Model 2), with a superior AUC of 0.810 (95% CI, 0.738–0.873; *p* < 0.001). (Table 5, Fig. 6). The model incorporating both clinical risk factors and LAAV% (Model 3) exhibited 74.3% sensitivity and 72.8% specificity, which indicates a balanced diagnostic performance for the hybrid approach with excellent calibration (Hosmer-Lemeshow [HL] *p* = 0.464). Restricted cubic spline (RCS) modeling (Fig. 7) revealed a J-shaped trajectory, with CMD risk remaining relatively stable at lower LAAV% levels, but increasing exponentially once LAAV% exceeded the tipping point of 41.7%. Furthermore, unsupervised clustering identified a distinct high-risk metabolic phenotype (Cluster 2) with a significantly higher CMD prevalence (67.2% vs. 42.6%, *p* = 0.005), characterized by elevated LAAV%, obesity, and dyslipidemia (**Supplementary Table 1**).

**Table 5.** **Comparison of ROC models for predicting coronary microvascular dysfunction**.

Models	AUC [95% CI]	Sensitivity (%)	Specificity (%)	Calibration (HL *p*)
Model 1 (LAAV%)	0.710 [0.630–0.791]	65.6	45.7	0.412
Model 2 (clinical factors)	0.756 [0.680–0.832]^ *^	50.0	79.7	0.395
Model 3 (Model 1 + 2)	0.810 [0.738, 0.873]^ †^	74.3	72.8	0.464

Note: Model 1: LAAV% only; Model 2: Clinical factors (age, sex, BMI, HbA1c, triglycerides, and smoking); Model 3: Hybrid model (Model 1 + Model 2). * *p* < 0.05 vs. Model 1; ^† ^*p* < 0.05 vs. Model 1 and Model 2 (based on the DeLong test). Abbreviations: AUC, area under the curve; CI, confidence interval; CMD, coronary microvascular dysfunction; HL, Hosmer-Lemeshow; LAAV, low-attenuation adipose volume; ROC, receiver operating characteristic.

## 4. Discussion

Most studies to date have focused on the total amount of EAT [24,25], previous research highlighted the qualitative composition of EAT as a superior marker for CMD [7,26]. Quantification of adipose size alone appears to be insufficient, and CCTA-derived adipose density may offer deeper insights into tissue characterization. In the present cohort, patients with preserved adipose volume still exhibited microvascular impairment if the EAT quality was “pathological” [27]. Utilizing an FFR cutoff of 0.75 ensured highly specific exclusion of flow-limiting epicardial disease—establishing a rigorous baseline for investigating isolated microvascular impairment, independent of borderline epicardial stenoses.

Increased LAAV may signify a pathophysiological sequence of adipocyte hypertrophy and hypoxia, promoting a pro-inflammatory transition [13,28,29]. Such expansion outpaces local perfusion [30,31], potentially recruiting M1 macrophages that secrete cytokines such as TNF-α and IL-6 [32]. Lacking a protective fascia, these EAT-derived mediators are hypothesized to infiltrate vessel walls via a ‘vasocrine effect’, potentially contributing to chronic microvascular injury. Conversely, fibrous and vascularized high-attenuation adipose tissue remains more stable and less detrimental to myocardial health.

Our data indicate that while other adipose components remained comparable between groups, LAAV% emerged as a superior marker for CMD [20]. Indeed, the total adipose increase in the CMD group was driven almost entirely by this low-density adipose, suggesting the local adipose environment rather than the total adipose amount may be largely responsible for damage to small vessels. In addition to releasing inflammatory signals, these hypertrophic adipocytes may leak excess free fatty acids (FFA), thereby potentially impairing nitric oxide (NO) production and contributing to chronic microvascular constriction from the outside in [33].

We propose two plausible mechanisms linking low-density adipose to CMD, with the first being biochemical and the second mechanical. First, without a protective layer, inflammation from M1 macrophages is hypothesized to seep into the vessels, which may lower the NO level and potentially cause the microvessels to remain constricted [34,35]. Second, EAT builds up in the tight space between the heart and its outer lining, potentially putting physical pressure on the small vessels. This could hypothetically squeeze and narrow the vessels, worsening blood flow to the heart muscle. The elevated pressure may contribute to increased IMR scores and limited CFR, suggesting the vessels simply lose their ability to expand under stress [36,37].

To investigate the independent predictive value of LAAV%, this study analyzed 155 patients. Standardized LAAV% emerged as a robust, independent predictor for CMD, even after adjusting for BMI, smoking history, and triglycerides within our parsimonious multivariable model [4,38]. This suggests that standardized LAAV% represents a more direct ‘metabolic footprint’ of epicardial adipose dysfunction than traditional systemic markers. While literature frequently highlights the impact of systemic glycemic markers on microvessels, our findings imply that localized adipose remodeling around the heart is a more proximal driver of microvascular impairment, making LAAV% a more specific biomarker in this cohort [9].

Furthermore, expanding beyond single-variable prediction, our unsupervised machine learning approach identified a distinct, high-risk metabolic phenotype (Cluster 2). This phenotype is characterized by elevated LAAV%, obesity, and dyslipidemia. The significantly higher prevalence of CMD in Cluster 2 (67.2% vs. 42.6%, *p* = 0.005, **Supplementary Table 1**) underscores the clinical utility of clustering for risk stratification. Although BMI and TGs showed high AUCs in identifying this phenotype, LAAV% remained a robust independent biomarker (AUC = 0.718, **Supplementary Fig. 1-3**), providing unique imaging evidence of the local inflammatory environment [14]. Current paradigms prioritize perivascular adipose phenotyping over traditional plaque imaging: a critical step toward elucidating microvascular health [15].

Our findings revealed a J-shaped relationship between LAAV% and CMD risk, with a biological tipping point at 41.7%. Beyond this threshold, ‘decompensated’ EAT expansion likely triggers a ‘vasocrine’ surge, potentially explaining the exponential rise in microvascular resistance. Incorporating LAAV% into routine CCTA evaluations could facilitate early identification and targeted intervention for high-risk INOCA patients before the occurrence of MACE.

The use of IMR and CFR was a key part of the current study, as these tools are the gold standard for defining CMD. This approach is highly accurate and helps to identify patients with open large arteries, but damaged small ones. The retrospective nature of this analysis prevents certain causal interpretations, and the possibility of lingering confounding effects remains a valid concern; nonetheless, our findings are consistent with the established ‘cross-talk’ between epicardial adipose and the vasculature, as well as the known role of M1 macrophages in driving microvascular impairment. By analyzing specific American Heart Association (AHA) segments, we can map out how the quality of localized adipose tissue correlates with regional blood flow deficits.

## 5. Limitations

Establishing causality remains challenging due to the study’s retrospective nature, particularly concerning the sequence of EAT remodeling and CMD development. Furthermore, the influence of unrecorded confounders such as systemic inflammation—may persist despite statistical adjustments. Finally, while bootstrapping ensured internal stability, multicenter external validation is required to generalize the LAAV% threshold of 41.7%.

## 6. Conclusions

Our findings demonstrate that the qualitative composition of EAT holds greater predictive significance for CMD than total volume alone. Specifically, standardized LAAV% serves as a robust and independent imaging biomarker for identifying microvascular dysfunction within a parsimonious multivariable framework. Our results suggest a plausible dual-mechanism model where hyper-inflammatory adipose tissue may exert both biochemical stress (via lower NO) and mechanical stress on the coronary microvasculature. The assessment of standardized EAT components via CCTA provides a valuable, non-invasive tool for identifying high-risk metabolic phenotypes, facilitating precision risk stratification and targeted intervention in CMD.

## Data Availability

Due to privacy-related restrictions regarding sensitive clinical imaging, the analyzed datasets remain confidential. However, the corresponding author can facilitate access to anonymized records for legitimate research endeavors upon justified request.

## Abbreviations

ANOCA, angina with non-obstructive coronary arteries; AUC, area under the curve; BMI, body mass index; BSA, body surface area; CAD, coronary artery disease; CCTA, coronary computed tomography angiography; CFR, coronary flow reserve; CI, confidence interval; CMD, coronary microvascular dysfunction; EAT, epicardial adipose tissue; EFD, epicardial adipose density; EFV, epicardial adipose volume; EFVi, epicardial adipose volume index; FBG, fasting blood glucose; FFA, free fatty acids; FFR, fractional flow reserve; FIB, fibrinogen; HAAV, high-attenuation adipose volume; HbA1c, glycated hemoglobin; HU, Hounsfield units; ICA, invasive coronary angiography; IMR, index of microcirculatory resistance; INOCA, Ischemia with non-obstructive coronary arteries; LAAV, low-attenuation adipose volume; LAAV%, percentage of low-attenuation adipose volume; LAAVi, low-attenuation adipose volume index; MACE, major adverse cardiovascular events; MINOCA, myocardial infarction with non-obstructive arteries; OR, odds ratio; RCS, restricted cubic spline; ROC, receiver operating characteristic; TG, triglycerides.
