# Supplementary material for: Percentage of Low-Attenuation Epicardial Adipose Volume (LAAV%) as an Independent Predictor of Coronary Microvascular Dysfunction: A Retrospective Cohort Study
Source: Rev Cardiovasc Med. 2026 Aug 5;27(8):50334. doi: 10.31083/RCM50334 (PMC13540017; doi:10.31083/RCM50334)
Supplement: Supplementary file 1 [file 2153-8174-27-8-50334-s1.zip › Supplementary Material.docx]

**Supplementary Materials: Phenotypic Clustering and Model Validation**

An unsupervised machine learning pipeline identified clinical and metabolic phenotypes associated with epicardial adiposity.

**Supplementary Fig. 1. K-means Clustering and Optimal Cluster Selection**

**Supplementary Fig. 1.** Unsupervised machine learning-based phenotypic clustering (*n* = 155). K-means algorithm partitioned the cohort using multidimensional features: EAT characteristics (LAAV%, HAAV%, EFVi) and metabolic markers (BMI, HbA1c, TG). Optimal cluster number (*k* = 2) followed elbow method and silhouette analysis. Spatial segregation distinguishes low-risk (Cluster 1) from high-risk (Cluster 2) phenotypes.

**Supplementary Fig. 2. PCA Loadings and Feature Importance**

**Supplementary Fig. 2.** PCA loading plot identifying primary phenotypic drivers. Vector direction and length represent individual variable contributions. Notably, "LAAV percent (LAAV%)" exhibits a dominant loading vector along Dim1—confirming its central role in characterizing the high-risk cluster. These results position LAAV% as a key "metabolic footprint" of adipose-cardiovascular crosstalk.

**Supplementary Fig. 3. Comparative Diagnostic Performance**

**Supplementary Fig. 3.** ROC analysis for Cluster 2 identification. Comparative evaluation of LAAV%, BMI, and TG. While metabolic markers (TG and BMI) show high diagnostic performance (AUC: 0.806 and 0.796), LAAV% remains a significant imaging biomarker (AUC: 0.718) for phenotype stratification. Data support integrating EAT subcomponents into traditional risk assessments to enhance high-risk CMD identification.

**Supplementary Table 1. Cluster Characterization**

| Variable | Cluster1 (*n* = 94) | Cluster2 (*n* = 61) | *P*-value |
| --- | --- | --- | --- |
| LAAV% | 0.46 ± 0.06 | 0.51 ± 0.06 | < 0.001 |
| EFVi (cm^3^/m^2^) | 42.45 ± 10.34 | 44.85 ± 8.16 | 0.111 |
| BMI (kg/m^2^) | 23.21 ± 2.09 | 25.85 ± 2.44 | < 0.001 |
| AGE (years) | 68.19 ± 9.3 | 60.13 ± 8.97 | < 0.001 |
| HbA1c (%) | 5.51 ± 0.59 | 6.25 ± 1.2 | < 0.001 |
| Triglycerides (mmol/L) | 1.42 ± 0.62 | 2.45 ± 1.17 | < 0.001 |
| LDL (mmol/L) | 2.01 ± 0.59 | 2.94 ± 0.73 | < 0.001 |
| Fibrinogen (g/L) | 2.83 ± 0.49 | 3.00 ± 0.53 | 0.039 |
| SEX (Male), *n* (%) | 54 (57.4%) | 34 (55.7%) | 0.965 |
| Hypertension, *n* (%) | 55 (58.5%) | 40 (65.6%) | 0.476 |
| Smoking history, *n* (%) | 15 (16.0%) | 15 (24.6%) | 0.262 |
| Myocardial bridge, *n* (%) | 29 (30.9%) | 22 (36.1%) | 0.617 |
| CMD, *n* (%) | 40 (42.6%) | 41 (67.2%) | 0.005 |

**Supplementary Table 1.** Clinical and imaging characteristics across machine learning-derived clusters. Cluster 2 represents a high-risk phenotype: characterized by elevated LAAV% and severe microvascular impairment (confirmed via invasive IMR/CFR). These findings align unsupervised phenotyping with clinical gold standards.
